# Improving stroke awareness through a culturally adapted audiovisual intervention in the United Arab Emirates

**DOI:** 10.3389/fneur.2025.1608381

**Published:** 2025-07-23

**Authors:** Michelle Cherfane, Jihan Safwan, Chadia Haddad, Hala Sacre, Pascale Salameh, Rawan Elkerenawy, Tala Abou El Kheir, Mariam Al Nuaimi, Leen Abou Mattar, Hassan Hosseini, Fouad Sakr, Katia Iskandar

**Affiliations:** ^1^Gilbert and Rose-Mary Chagoury School of Medicine, Lebanese American University, Byblos, Lebanon; ^2^College of Health Sciences, Abu Dhabi University, Abu Dhabi, United Arab Emirates; ^3^Institut National de Santé Publique, d’Épidémiologie Clinique et de Toxicologie (INSPECT-LB), Beirut, Lebanon; ^4^School of Pharmacy, Lebanese International University, Beirut, Lebanon; ^5^Psychiatric Hospital of the Cross, Beirut, Lebanon; ^6^Modern University for Business and Science, Beirut, Lebanon; ^7^Order of Pharmacists of Lebanon, Beirut, Lebanon; ^8^Faculty of Public Health, Lebanese University, Fanar, Lebanon; ^9^University of Nicosia Medical School, Nicosia, Cyprus

**Keywords:** stroke-related knowledge, audiovisual intervention, health literacy, public health education, United Arab Emirates

## Abstract

**Objectives:**

This study evaluates the effectiveness of a brief, culturally tailored educational video in improving stroke-related knowledge among residents of the United Arab Emirates (UAE).

**Methods:**

A pre-post intervention study was conducted with 407 UAE residents aged 25 years and older. Participants viewed a 3-min educational video addressing stroke symptoms, risk factors, and preventive strategies. Stroke knowledge was measured using a structured questionnaire immediately before and after the video. Statistical analyses included paired t-tests, repeated measures ANOVA, and linear regression models.

**Results:**

Stroke knowledge significantly increased following the intervention (mean score: 20.80 pre-test to 23.53 post-test; *p* < 0.001), with notable improvements in identifying symptoms and risk factors. Regression analyses indicated that female gender, higher education, and healthy lifestyle practices positively influenced knowledge gains, whereas older age was associated with smaller improvements.

**Conclusion:**

A brief, culturally relevant audiovisual intervention effectively enhances stroke-related knowledge. Such scalable educational tools should be integrated into global public health strategies to promote earlier stroke recognition and intervention.

## Introduction

Stroke remains a leading global cause of death and long-term disability, significantly impacting public health worldwide (1). The increasing burden of stroke is particularly pronounced in rapidly urbanizing countries, such as the United Arab Emirates (UAE), which has experienced dramatic economic growth and improved living standards over recent decades (2). This rapid socioeconomic transition has been accompanied by significant shifts in lifestyle, characterized by increased sedentary behaviors, unhealthy diets, and consequently heightened prevalence of risk factors for non-communicable diseases including obesity, diabetes mellitus, and cardiovascular diseases (3, 4).

In the Middle Eastern region, especially in the UAE, the incidence of stroke has risen sharply due to demographic transitions, notably an aging population, and widespread adoption of lifestyle behaviors conducive to chronic disease development (5). Despite these increasing risks, public awareness and recognition of stroke symptoms and associated risk factors remain limited, often leading to delayed medical intervention and suboptimal clinical outcomes (6).

Evidence suggests that targeted educational interventions—especially brief, audiovisual tools—can significantly improve public knowledge of stroke. For example, a recent interventional study demonstrated that a short video-based tool effectively enhanced stroke-related knowledge among lay populations (7). Similarly, structured public health interventions, such as campaigns employing the FAST acronym (Facial drooping, Arm weakness, Speech difficulties, and Time to call emergency services), have demonstrated effectiveness in improving stroke symptom recognition and facilitating quicker medical response (8).

Extensive research underscores the importance of enhancing public knowledge as a vital component of stroke prevention and management, especially among populations at higher risk (9, 10). Effective public health messaging can significantly influence individual behaviors, encourage preventive practices, and reduce the time taken to seek emergency care, thereby decreasing stroke-related morbidity and mortality (11–14).

The current study aims to evaluate the impact of a culturally tailored educational video intervention designed specifically for the UAE population. It seeks to measure changes in public awareness regarding stroke symptoms, risk factors, and preventive strategies, assessing knowledge before and after exposure to the intervention. This study does not assess long-term retention or behavioral change, which would require more extensive longitudinal or community-based designs. Nonetheless, the findings offer insight into the potential value of brief, scalable audiovisual education tools for enhancing stroke preparedness in rapidly developing settings.

## Methods

### Study design

This study utilized a pre-post intervention approach without a control group, conducted from July to September 2022. The primary aim was to assess the impact of a culturally tailored educational video on public awareness regarding stroke, while also examining demographic and lifestyle factors that could influence the effectiveness of the intervention.

### Participants

The study population included adults aged 25 years and older residing in the UAE. This specific demographic was chosen because adulthood marks a critical period when lifestyle behaviors solidify, and many stroke risk factors start to develop, making it an ideal time frame for effective preventive educational interventions (15). Participants were recruited using various social media platforms, including WhatsApp, Instagram, Twitter, and Facebook, to ensure wide demographic reach. Eligibility criteria included: age ≥ 25 years, residence in the UAE, and provision of informed electronic consent. Individuals with formal prior stroke training or cognitive impairment were excluded.

### Ethical considerations

Ethical approval for this study was obtained from the Institutional Review Board at [Blinded for review] (approval code CoHS–22-05-00018, dated May 16, 2022). All study procedures complied with the ethical standards outlined in the Declaration of Helsinki. Participants provided informed consent electronically by confirming their understanding of the study objectives and voluntarily agreeing to participate. They were informed of their right to withdraw at any point without any repercussions and were not offered financial incentives.

### Sample size calculation

A sample size calculation was performed using G-Power software (version 3.0.10). With an anticipated small effect size (f^2^ = 0.0526, corresponding to an R^2^ = 0.05) and accounting for up to 15 predictors within multiple regression analyses, a minimum of 371 participants was required to achieve 80% statistical power at an alpha level of 0.05. To allow for potential participant drop-out or incomplete responses, the target sample size was set at approximately 400 participants.

### Online survey instrument

The survey instrument was developed using Google Forms and featured a 3-min educational video titled “Think Fast, Act Fast to Save a Life,” aligned with educational standards set by the American Stroke Association. To ensure linguistic and cultural appropriateness, both the questionnaire and video underwent rigorous translation processes based on World Health Organization guidelines (16), including forward translation to Arabic and back-translation to English. Any discrepancies encountered were resolved collaboratively among the translators and the principal investigator. A panel of bilingual stroke and public health experts reviewed all materials for accuracy and cultural suitability.

The survey comprised six sections: (1) demographic details, (2) lifestyle behaviors, (3) self-reported health status, (4) stroke knowledge pre-test (assessing awareness of risk factors, symptoms, and prevention measures), (5) viewing of the educational video (offered in English and Arabic), and (6) stroke knowledge post-test, repeating the pre-test questions.

The stroke knowledge test included 30 items assessing awareness of stroke symptoms, risk factors, prevention, and treatment. The questionnaire was validated through expert review for face and content validity. A pilot test with 20 participants confirmed clarity and usability. A 3-point increase equated to a 10% gain in correct responses, deemed meaningful for a brief, single-session intervention.

### Video content and development

The 3-min video titled *“*Think FAST, Act FAST to Save a Life*”* was developed based on evidence-based guidelines from the American Stroke Association and WHO stroke education resources. It included visual and audio content addressing stroke warning signs (FAST acronym), key risk factors (e.g., hypertension, diabetes, smoking), preventive measures (e.g., healthy diet, physical activity), and emergency response actions. The script and visuals were adapted for cultural relevance by bilingual healthcare professionals, using regionally familiar examples and language.

### Recruitment and distribution

Participants were recruited through various social media platforms (WhatsApp, Instagram, Twitter, and Facebook) to ensure broad demographic representation. The survey and accompanying video links were disseminated electronically in both English and Arabic. While the UAE is culturally diverse, this intervention was specifically designed for Arabic-speaking adults residing in the country. The content reflected cultural and linguistic norms common among Arab populations in the UAE. Full access to these materials is provided in Supplementary material 1.

### Statistical analysis

All statistical analyses were performed using SPSS software version 25. Descriptive statistics summarized categorical data as frequencies and percentages, and continuous variables as means and standard deviations (SD). Normality of the distribution for dependent variables (total knowledge scores) was confirmed by assessing skewness and kurtosis, which fell within acceptable ranges (−2 to +2).

Pre- and post-intervention stroke knowledge scores were compared using paired sample t-tests. Repeated measures ANOVA was used to evaluate changes in knowledge scores after adjusting for covariates, including age, gender, marital status, smoking habits, alcohol consumption, education level, healthy lifestyle indicators, history of stroke, family stroke history, and existing medical conditions.

Three linear regression models were conducted: (1) predicting baseline knowledge, (2) predicting post-intervention knowledge, and (3) identifying predictors of knowledge score change (changes from pre- to post-intervention). Predictor variables included demographic characteristics and stroke-related risk factors. Statistical significance was established at a *p*-value of less than 0.05.

## Results

### Participant characteristics and stroke risk profile

The study involved 407 participants, primarily females (53.6%), married individuals (55.8%), and university-educated adults (68.3%), with a majority aged between 25 and 34 years (50.9%). Approximately one-quarter of the respondents were healthcare professionals (25.6%). Key stroke risk factors identified among participants included a previous stroke event in 3.2% and a positive family history of stroke in 31%. Health conditions prevalent in the sample included severe headaches or migraines (22.3%), hypercholesterolemia (17.5%), and hypertension (17.2%). Lifestyle-related risks showed notable proportions of current smokers (25.6%), alcohol consumers (13.0%), and participants predominantly engaging in mild physical activity (52.8%). Dietary patterns indicated that the majority regularly consumed vegetables (79.4%), dairy products (75.2%), and legumes (74.9%). The mean household crowding index was 1.43 ± 0.78, and the average stress score according to the Beirut Distress Scale (17) was 8.91 ± 6.40 (Table 1).

**Table 1:** Sociodemographic characteristics of the participants and stroke risk factors (*N* = 407).

Variable	*N* (%)
Gender	
Male	189 (46.4%)
Female	218 (53.6%)
Age	
25–34	207 (50.9%)
35–44	110 (27.0%)
45–54	65 (16.0%)
55 and above	25 (6.1%)
Marital status	
Single/widowed/divorced	180 (44.2%)
Married	227 (55.8%)
Education level	
Primary	11 (2.7%)
Secondary	38 (9.3%)
University	358 (88%)
Being a healthcare professional	
Yes	104 (25.6%)
No	303 (74.4%)
	Mean ± SD
Household crowding index	1.43 ± 0.78
Stroke risk factors (*N* = 407)	
Family history of stroke	
Yes	126 (31.0%)
No	281 (69.0%)
Previous stroke	
Yes	13 (3.2%)
No	394 (96.8%)
Smoking	
Never smoke	276 (67.8%)
Current smoker	104 (25.6%)
Former smoker (quit more than 6 months ago)	27 (6.6%)
Alcohol	
Do not drink	345 (84.8%)
On weekends	55 (13.5%)
Occasionally	7 (1.7%)
Physical activity	
Not applicable	24 (5.9%)
Mild	215 (52.8%)
Moderate	124 (30.5%)
Vigorous	44 (10.8%)
BMI	
Underweight	14 (3.4%)
Healthy weight	179 (44.0%)
Overweight	134 (32.9%)
Obese	80 (19.7%)
Having any of the following health conditions	
High blood pressure	70 (17.2%)
History of myocardial infarction	17 (4.2%)
Family history of myocardial infarction	50 (12.3%)
History of coronary artery disease	27 (6.6%)
High cholesterol level	71 (17.5%)
Diabetes	56 (13.8%)
History of deep vein thrombosis	14 (3.4%)
History of pulmonary embolism	13 (3.2%)
Severe headache (migraine)	90 (22.3%)
	Mean ± SD
BDS total	8.91 ± 6.40

BMI, Body Mass Index; BDS, Beirut Distress Scale.

### Effectiveness of educational intervention on stroke-related knowledge

The educational intervention resulted in statistically significant improvements across nearly all stroke knowledge areas (Table 2). Notably, participants demonstrated marked improvement in recognizing stroke symptoms, identifying key risk factors, and understanding preventive actions. After adjusting for relevant sociodemographic and health-related variables—including age, gender, marital status, education level, household crowding, lifestyle habits, and medical history—the analysis confirmed a significant increase in overall stroke knowledge, rising from an average pre-intervention score of 20.80 to 23.53 post-intervention (*p* < 0.001), underscoring the intervention’s effectiveness (Table 3).

**Table 2:** Variation of the stroke knowledge pre and post education session.

Knowledge items	Pre	Post	*p*-value
	Mean ± SD	Mean ± SD	
Knowledge total score	20.80 ± 8.58	23.53 ± 8.16	<0.001
Stroke is a serious condition that occurs in the brain	0.74 ± 0.43	0.78 ± 0.40	0.031
Types of stroke			
Stroke occurs when the arteries in the brain are partially or completely blocked	0.69 ± 0.46	0.79 ± 0.40	<0.001
Stroke occurs when the brain arteries bleed	0.47 ± 0.49	0.65 ± 0.47	<0.001
Risk score	7.35 ± 3.83	8.67 ± 3.51	<0.001
The risk of stroke increases when uncontrolled blood pressure	0.74 ± 0.43	0.84 ± 0.35	<0.001
The risk of stroke increases when irregular heart rhythm	0.58 ± 0.49	0.75 ± 0.43	<0.001
The risk of stroke increases when obesity	0.62 ± 0.48	0.77 ± 0.42	<0.001
The risk of stroke increases when diabetes mellitus	0.54 ± 0.49	0.72 ± 0.44	<0.001
The risk of stroke increases when heart diseases	0.69 ± 0.46	0.77 ± 0.41	<0.001
The risk of stroke increases when you smoke	0.76 ± 0.42	0.85 ± 0.35	<0.001
The risk of stroke increases when drinking alcohol	0.73 ± 0.44	0.84 ± 0.36	<0.001
The risk of stroke increases when lack of regular exercise	0.72 ± 0.44	0.82 ± 0.37	<0.001
The risk of stroke increases when stress	0.77 ± 0.41	0.83 ± 0.37	0.002
Warning signs	4.07 ± 2.29	4.75 ± 2.07	<0.001
warning sign of stroke unable to speak properly	0.75 ± 0.43	0.85 ± 0.35	<0.001
warning sign of stroke eye falling downward	0.61 ± 0.48	0.75 ± 0.43	<0.001
warning sign of stroke paralysis in the arm or leg or both	0.72 ± 0.44	0.81 ± 0.39	<0.001
warning sign of stroke dizziness	0.64 ± 0.47	0.77 ± 0.41	<0.001
warning sign of stroke severe headache	0.66 ± 0.47	0.77 ± 0.41	<0.001
warning sign of stroke not being able to answer questions	0.65 ± 0.47	0.79 ± 0.40	<0.001
Prevention measures	5.32 ± 1.73	5.45 ± 1.61	0.028
To reduce the risk of stroke you need to eat healthy food	0.90 ± 0.29	0.91 ± 0.28	0.346
To reduce the risk of stroke you need to exercise regularly	0.89 ± 0.30	0.92 ± 0.26	0.050
To reduce the risk of stroke you need to avoid stress	0.87 ± 0.32	0.90 ± 0.28	0.023
To reduce the risk of stroke you need to maintain a healthy weight	0.88 ± 0.31	0.89 ± 0.30	0.346
To reduce the risk of stroke you need to take your medications on time	0.87 ± 0.33	0.91 ± 0.28	0.002
To reduce the risk of stroke you need to follow-up with your doctor	0.88 ± 0.31	0.89 ± 0.30	0.318
Treatment of stroke			
How stroke is treated by medications	0.72 ± 0.44	0.83 ± 0.37	<0.001
How stroke is treated by rehabilitation	0.59 ± 0.49	0.74 ± 0.43	<0.001
Acting fast in the case of stroke			
Call an ambulance straight away	0.84 ± 0.36	0.84 ± 0.36	0.746

**Table 3:** Public knowledge of stroke among UAE participants.

Question	Pre-education	Post-education	*p*-value
	*n* (%)	*n* (%)	
Definition of stroke (1 correct answer: brain)			
Correct	303 (74.4%)	321 (78.9%)	<0.001
Wrong	60 (14.7%)	39 (9.6%)	<0.001
Unknown	44 (10.8%)	47 (11.5%)	<0.001
Types of stroke (2 types)			
1 type correct (ischemic or hemorrhagic)	295 (72.5%)	332 (81.6%)	<0.001
2 types correct (ischemic and hemorrhagic)	179 (44.0%)	257 (63.1%)	<0.001
Unknown	121 (29.7%)	69 (17.0%)	<0.001
Risk factors for stroke—Diseases (Overall 5 factors)			
At least 1 factor correct	106 (26.0%)	70 (17.2%)	<0.001
2 factors correct	32 (7.9%)	18 (4.4%)	<0.001
3 factors correct	44 (10.8%)	25 (6.1%)	<0.001
More than 3 factors correct	225 (55.3%)	294 (72.2%)	<0.001
Unknown	139 (34.2%)	77 (18.9%)	<0.001
Risk factors for stroke—Lifestyle and others (Overall 6 factors)			
At least 1 factor correct	85 (20.9%)	56 (13.8%)	<0.001
2 factors correct	24 (5.9%)	15 (3.7%)	<0.001
3 factors correct	42 (10.3%)	18 (4.4%)	<0.001
More than 3 factors correct	256 (62.9%)	318 (78.1%)	<0.001
Unknown	87 (21.4%)	48 (11.8%)	<0.001
Stroke warning signs (overall 5 symptoms)			
At least 1 symptom correct	86 (21.1%)	57 (14.0%)	<0.001
2 symptoms correct	19 (4.7%)	5 (1.2%)	<0.001
3 symptoms correct	25 (6.1%)	18 (4.4%)	<0.001
More than 3 symptoms correct	277 (68.1%)	327 (80.3%)	<0.001
Unknown	151 (37.1%)	80 (19.7%)	<0.001
Stroke Treatment (overall 2 treatment options)			
1 Treatment option correct (meds or rehab)	311 (76.4%)	351 (86.2%)	<0.001
2 Treatment options correct (meds and rehab)	227 (55.8%)	292 (71.7%)	<0.001
In case of stroke, when to call ambulance (RIGHT AWAY)			
Correct	342 (84.0%)	344 (84.5%)	<0.001
Wrong	65 (16.0%)	63 (15.5%)	<0.001
Prevention of stroke (overall 6 lifestyle measures)			
At least 1 measure correct	37 (9.1%)	32 (7.9%)	<0.001
2 measures correct	3 (0.7%)	3 (0.7%)	<0.001
3 measures correct	7 (1.7%)	4 (1.0%)	<0.001
More than 3 measures correct	360 (88.5%)	368 (90.4%)	<0.001

### Bivariate associations with stroke knowledge scores

Bivariate analysis identified several factors significantly associated with stroke knowledge scores at baseline and post-intervention. Higher pre-intervention scores correlated with being female, aged 45–54, single status, presence of a family history of stroke, non-smoking status, alcohol consumption, and engagement in a healthy lifestyle. Post-intervention analysis similarly highlighted higher knowledge scores among females, individuals with university education, and those with chronic high-risk medical conditions (*p* < 0.001 for all comparisons) (Table 4).

**Table 4:** Factors affecting the knowledge score (pre/post-test).

Variable	Knowledge score			
	Pre-test	*p* value	Post-test	*p* value
Gender				
Male	19.80 ± 9.31	0.03	22.64 ± 8.98	0.043
Female	21.66 ± 7.81		24.30 ± 7.31	
Age (years)				
25–34				
No	22.01 ± 7.86	0.005	24.85 ± 6.92	0.001
Yes	19.64 ± 9.09		22.26 ± 9.05	
35–44				
No	20.53 ± 8.71	0.286	23.14 ± 8.44	0.114
Yes	21.55 ± 8.21		24.58 ± 7.29	
45–54				
No	20.47 ± 8.73	0.049	23.11 ± 8.46	0.003
Yes	22.57 ± 7.57		25.74 ± 5.94	
55 and above				
No	20.69 ± 8.66	0.282	23.52 ± 8.21	0.905
Yes	22.60 ± 7.08		23.82 ± 7.56	
Marital status				
Single/widowed/divorced	19.73 ± 9.17	0.27	22.47 ± 9.08	0.22
Married	21.66 ± 8.00		24.37 ± 7.27	
University degree				
No	19.29 ± 9.64	0.073	21.08 ± 9.87	0.004
Yes	21.27 ± 8.19		24.27 ± 7.43	
Being a healthcare professional				
No	20.39 ± 8.34	0.09	23.32 ± 8.12	0.375
Yes	22.04 ± 9.17		24.14 ± 8.31	
Previous stroke				
No	20.87 ± 8.53	0.441	23.57 ± 8.15	0.632
Yes	19.00 ± 10.16		22.46 ± 8.93	
Family history of stroke				
No	19.98 ± 8.94	0.002	22.83 ± 8.77	0.004
Yes	22.64 ± 7.42		25.09 ± 6.39	
Any high-risk disease				
No	20.51 ± 8.82	0.297	22.92 ± 8.89	0.039
Yes	21.40 ± 8.07		24.54 ± 6.78	
Current smoker				
No	21.33 ± 8.53	0.033	23.89 ± 8.10	0.132
Yes	19.26 ± 8.58		22.49 ± 8.30	
Consumes alcohol				
No	20.33 ± 8.79	<0.001	23.22 ± 8.46	0.007
Yes	24.02 ± 6.19		25.62 ± 6.44	
Healthy lifestyle				
No	20.36 ± 8.79	0.006	23.11 ± 8.52	0.002
Yes	23.14 ± 7.03		25.68 ± 5.58	

### Multivariable predictors of stroke knowledge improvements

Multivariable linear regression analyses explored predictors influencing knowledge scores both pre- and post-intervention, and factors affecting knowledge gains between these time points. Initially, higher baseline stroke knowledge scores were significantly associated with female gender, family history of stroke, alcohol consumption, and healthier lifestyle practices (Table 5, Model 1). Post-intervention results reinforced these findings, additionally highlighting the positive influence of having a university education (Table 5, Model 2). Conversely, older age (≥55 years) and alcohol use negatively influenced the magnitude of knowledge gains following the intervention, suggesting targeted educational adjustments might be necessary for these subgroups (Table 5, Model 3). These findings have substantial public health implications, supporting the efficacy of targeted educational interventions to significantly improve community awareness of stroke, particularly in urbanizing and diverse settings.

**Table 5:** Multivariable analysis.

Independent variables	Unstandardized beta	Standardized beta	*p*-value	Confidence interval	
				Lower bound	Upper bound
Model 1: Taking the knowledge of stroke pre-test as the dependent variable					
Age in years (25-34)	−3.302	−1.737	0.083	−7.040	0.436
Age in years (35-34)	−3.237	−1.716	0.087	−6.944	0.471
Age in years (45–54)	−1.170	−0.601	0.548	−5.000	2.659
Gender (female vs. male^*^)	2.200	2.388	0.017	0.389	4.011
Marital status (married vs. single^*^)	1.521	1.565	0.119	−0.390	3.432
History of stroke (yes vs. no^*^)	−4.554	−1.880	0.061	−9.317	0.210
Family history of stroke (yes vs. no^*^)	2.067	2.208	0.028	0.226	3.909
Alcohol (yes vs. no^*^)	4.674	3.624	<0.001	2.138	7.209
Healthcare professional (yes vs. no^*^)	1.486	1.506	0.133	−0.453	3.425
Current smoker (yes vs. no^*^)	−0.719	−0.722	0.471	−2.675	1.237
Any high-risk disease (yes vs. no^*^)	−0.153	−0.174	0.862	−1.884	1.578
Healthy lifestyle (yes vs. no^*^)	2.700	2.414	0.016	0.501	4.899
University degree (yes vs. no^*^)	1.349	1.339	0.181	−0.631	3.329
Model 2: Taking the knowledge of stroke post-test as the dependent variable					
Age in years (25-34)	−1.367	−0.756	0.450	−4.923	2.189
Age in years (35-34)	−0.660	−0.368	0.713	−4.187	2.867
Age in years (45–54)	1.077	0.581	0.561	−2.566	4.720
Gender (female vs. male^*^)	1.788	2.041	0.042	0.066	3.511
Marital status (Married vs. single^*^)	1.217	1.317	0.189	−0.601	3.035
History of stroke (yes vs. no^*^)	−2.684	−1.165	0.245	−7.215	1.847
Family history of stroke (yes vs. no^*^)	1.576	1.769	0.078	−0.176	3.327
Alcohol (yes vs. no^*^)	3.181	2.594	0.010	0.770	5.593
Healthcare professional (yes vs. no^*^)	0.370	0.395	0.693	−1.474	2.215
Current smoker (yes vs. no^*^)	−0.210	−0.222	0.825	−2.071	1.651
Any high-risk disease (yes vs. no^*^)	0.774	0.924	0.356	−0.872	2.421
Healthy lifestyle (yes vs. no^*^)	2.268	2.131	0.034	0.176	4.359
University degree (yes vs. no^*^)	2.383	2.487	0.013	0.499	4.266
Model 3: Taking the pre-test and post-test difference in knowledge as the dependent variable					
Gender Females vs. Males^*^	−0.411	−0.037	0.518	−1.661	0.838
Age 35–44 vs. 25–34 years^*^	−0.641	−0.057	0.403	−2.145	0.863
Age 45–54 vs. 25–34 years^*^	−0.329	−0.022	0.715	−2.103	1.444
Age 55 above vs. 25–34 years^*^	−2.577	−0.111	0.048	−5.135	−0.018
University degree	1.034	0.077	0.138	−0.332	2.400
Marital status 2 vs. 1^*^	−0.303	−0.027	0.651	−1.622	1.015
Ever had stroke	1.870	0.059	0.264	−1.417	5.157
Relative had stroke	−0.492	−0.041	0.447	−1.762	0.779
Alcohol consumption	−1.493	−0.090	0.094	−3.242	0.257
Healthcare professional	−1.116	−0.086	0.102	−2.454	0.223
Current smoker	0.509	0.040	0.459	−0.841	1.859
Any high risk of disease	0.927	0.082	0.128	−0.267	2.122
Healthy LIFESTYLE INDEX	−0.432	−0.028	0.576	−1.950	1.085

Variables entered in the two models: gender, age, ever stroke, History of stroke, healthcare professional, smoking, alcohol, healthy lifestyle, medical illness, marital status and level of education. ^*^Reference group.

## Discussion

This study assessed the impact of a culturally adapted video-based educational intervention on stroke-related knowledge in the UAE population. The intervention notably improved knowledge regarding stroke risk factors, warning symptoms, and preventive practices. Significantly, this research marks the first nationwide effort within the UAE to utilize audiovisual materials specifically for public stroke education, providing an effective, scalable, and engaging alternative to traditional educational methods such as printed materials and face-to-face sessions (18). Similarly, a culturally adapted stroke prevention program delivered via a mobile app on WeChat in China showed high usability and participant satisfaction, emphasizing the potential of mobile platforms for culturally relevant stroke education (19). These findings are consistent with previous research, underscoring video interventions’ effectiveness in enhancing health literacy and immediate patient outcomes across diverse populations (20–22). Similar outcomes were observed in heart failure patients, where a digital education intervention (HF-DEM) significantly improved quality of life, supporting the effectiveness of tailored digital tools across various health conditions (23).

Participants notably improved their ability to distinguish between stroke and heart attack symptoms post-intervention, a critical factor in ensuring prompt and appropriate medical responses, aligning with findings from international studies (24–26). Moreover, the study demonstrated a substantial increase in awareness of stroke symptoms, notably the FAST criteria (Facial drooping, Arm weakness, Speech difficulties, Time to call emergency services), aligning with global evidence supporting targeted multimedia educational efforts (27–29). Additionally, consistent high willingness among participants to seek immediate medical attention for stroke symptoms reflects successful communication of the urgency and severity associated with strokes, corroborating previous research (30, 31). Beyond awareness and prevention, digital tools have also shown promise in stroke rehabilitation, with meta-analytic findings supporting online management systems for improving early physical activity during post-stroke recovery (32). Nevertheless, this study identified gaps in public understanding regarding specific stroke treatments (33), echoing earlier research emphasizing the importance of educating the public about timely intervention strategies (34).

Baseline stroke prevention knowledge among participants was relatively high and further improved following the intervention (35–37). The comparatively higher initial knowledge levels observed may reflect the participants’ educational backgrounds and overall health literacy, aligning with other studies highlighting correlations between educational attainment and stroke prevention knowledge (38–40).

Consistent with prior research, females demonstrated superior stroke knowledge, likely attributable to heightened health awareness and interest in preventive healthcare topics among women (41–43). Although direct evidence linking alcohol consumption to stroke knowledge remains limited, findings suggest regular alcohol consumers may exhibit increased health awareness or heightened risk perception, prompting greater information-seeking behaviors (44). Additionally, participants with family stroke histories displayed greater baseline knowledge, reinforcing findings from Nigeria and Morocco, and highlighting the importance of personal experiences and interpersonal communications in disseminating health information (45–47).

Health-conscious behaviors, such as regular physical activity (48) and nutritious diets (49), correlated positively with enhanced stroke knowledge, suggesting health-oriented individuals might proactively seek health information (50). Conversely, older adults (aged ≥55) exhibited modest knowledge improvements post-intervention despite higher stroke risk due to prevalent comorbidities such as hypertension and diabetes (51). The weaker improvement among older adults and marginal significance associated with alcohol consumption suggest targeted interventions could further improve awareness and preventive actions within these groups.

Collectively, recent evidence underscores a growing global shift toward digital, scalable, and culturally relevant education tools as viable strategies to reduce stroke burden and health disparities.

### Implications for practice

The study outcomes emphasize transitioning to audiovisual health education resources as highly beneficial for public health programs targeting stroke prevention. Comprehensive, multimedia-driven campaigns incorporating local medical expertise and using both traditional and digital platforms could effectively raise awareness, correct misconceptions, and expedite medical responses. Adopting evidence-based multimedia interventions may significantly enhance stroke outcomes by ensuring timely recognition and management. Moreover, incorporating brief, evidence-based audiovisual interventions into national stroke education campaigns may help overcome traditional literacy, accessibility, and resource barriers, particularly in underserved or multiethnic populations. These tools can also serve as valuable components in national stroke registries and prevention frameworks to track awareness trends and tailor interventions accordingly.

### Limitations

This study has several limitations. First, the online survey design may have introduced selection bias, favoring younger, digitally literate individuals—many with healthcare backgrounds—which limits generalizability to the broader UAE population, particularly older adults or those with limited access to technology. Second, the absence of a control group limits causative conclusions about knowledge improvements exclusively attributable to the video intervention. Additionally, using closed-ended questions might have amplified perceived knowledge levels. Moreover, the cross-sectional design precluded assessing long-term retention and behavioral changes, necessitating future longitudinal research. Finally, multiple statistical comparisons were performed in this analysis, increasing the risk of Type I error; future studies should consider adjusting for this or limiting subgroup analyses to predefined hypotheses.

## Conclusion

In conclusion, these findings highlight the effectiveness of audiovisual interventions in improving public stroke literacy, representing a feasible, cost-efficient, and engaging approach for diverse populations. Future public health strategies should broadly implement multimedia educational initiatives to facilitate meaningful community engagement, enhance preventive behaviors, and ultimately reduce stroke-related morbidity and mortality.

## Data Availability

The raw data supporting the conclusions of this article will be made available by the authors, without undue reservation.
