# Supplementary material for: Improving stroke awareness through a culturally adapted audiovisual intervention in the United Arab Emirates
Source: Front Neurol. 2025 Jul 23;16:1608381. doi: 10.3389/fneur.2025.1608381 (PMC12325011; doi:10.3389/fneur.2025.1608381)
Supplement: Supplementary file 1 [file Table_1.docx]

**Supplementary File 1**

**Procedure**

The questionnaire was available in both languages and was electronically distributed using Google

Forms and can be accessed using the following link: <https://forms.gle/Xr3U45Wwuee9imB36>

The questionnaire included a three-minute educational video available in both English and Arabic.

The English version can be accessed at <https://youtu.be/uzN-Q5SUKmA>, while the Arabic version is available at <https://youtu.be/yEvvQlX37gs>.
